# Clinical Characteristics, Complications, and Predictors of Poor Outcome Among Hospitalized Adult COVID-19 Patients: A Retrospective Cohort Study

**DOI:** 10.7759/cureus.28953

**Published:** 2022-09-08

**Authors:** Tariq Abdulrahman Tabbakh, Hashem H Alhashemi, Khalid Alharbi, Sultan Qanash, Mohammed S Alzahrani, Ahmed Saati, Samaher Alsulami, Atheer Alsulami, Alfaisal Neyazi, Abdullah Alzahrani, Ziad A Taher, Ghala Aljedaani, Abdulkareem Alhejaili

**Affiliations:** 1 Medicine, Ministry of National Guard-Health Affairs, Jeddah, SAU; 2 Medicine, King Saud bin Abdulaziz University for Health Sciences, Jeddah, SAU; 3 Medicine, King Abdulaziz Medical City, Jeddah, SAU

**Keywords:** predictors, outcome, updated charlson comorbidity index, intensive care unit, covid-19

## Abstract

Background

Many international studies have reported the outcomes and predictors of coronavirus disease 2019 (COVID-19); however, only a few national studies have reported predictors of poor outcomes among adult hospitalized patients with COVID-19. Therefore, this study aimed to describe the clinical characteristics and complications of COVID-19 and identify predictors of poor outcomes.

Methods

This was a retrospective cohort study. All adult patients confirmed with COVID-19 who were admitted at the King Abdulaziz Medical City (KAMC)-Jeddah between March 1, 2020, and December 31, 2020, were included; pediatric and pregnant patients were excluded. The clinical features and complications of COVID-19 were tested for association with poor outcomes (intensive care unit [ICU] admission or death) using chi-square and Fisher’s exact tests. In addition, logistic regression analysis was performed to identify the predictors of poor outcomes.

Results

A total of 527 patients were included in this study. Forty-two patients (8%) (6-10, 95% confidence interval [CI]) died: 13 in the general wards and 29 in the ICU. Of the 84 patients admitted to the ICU, 65 underwent invasive mechanical ventilation. Poor outcome affected 97 patients (18%) (15-22, 95% CI). Shortness of breath, oxygen saturation <92%, and abnormal chest x-ray findings were associated with poor outcomes (P-value < 0.001). In addition, lymphocyte counts were significantly lower, while c-reactive protein levels were significantly higher among patients with poor outcomes (P-value < 0.001). The most common complications were acute cardiac (83 patients, 16%), acute kidney (78 patients, 15%), and liver injuries (76 patients, 14%). Predictors of poor outcome were the updated Charlson comorbidity index (CCI) (odds ratio [OR] 1.2 [95% CI 1.1-1.4]), liver injury (OR 2.6 [95% CI 1.3-4.9]), acute kidney injury (OR 4.3 [95% CI 2.3-7.8]), and acute cardiac injury (OR 5.1 [95% CI 2.8-9.4]).

Conclusions

COVID-19 disease is associated with significant morbidity and mortality. Predictors of poor outcomes among COVID-19 hospitalized patients were the updated CCI, liver injury, acute kidney, and acute myocardial injuries. Subsequently, the risk of poor COVID-19 outcomes is increased among patients with multiple comorbidities and/or multiple COVID-19 complications.

## Introduction

The coronavirus disease 2019 (COVID-19) pandemic started in China in December 2019 [1]. The first COVID-19 case in Saudi Arabia was reported in March 2020 [2]. According to the Saudi Ministry of Health website (accessed April 28th, 2022), the total number of cases since the onset of the pandemic is 753,822 with 9,085 reported deaths due to COVID-19 infection [3]. Although COVID-19 published research has expanded globally, there is a relative deficiency in COVID-19 original research nationally [4]. Few national studies have yielded descriptive and basic univariate analysis results in the early phase of the pandemic regarding COVID-19's clinical features and outcomes [5-9]. Later, a few more retrospective cohorts reported results of multivariable regression models that predicted risk factors for mortality and the critical course of disease among patients with COVID-19 [10-13].

This study aimed to describe the clinical characteristics and complications of COVID-19. In addition, predictors of intensive care unit (ICU) admission and death among adult hospitalized patients with COVID-19 were identified.

## Materials and methods

This retrospective cohort study was approved by the institutional review board of the King Abdullah International Medical Research Center. Informed consent was waived due to the retrospective design of the study. All adult patients admitted to the general medical wards and ICUs with positive reverse transcription polymerase chain reaction (RT-PCR) test results for COVID-19 at the KAMC-Jeddah between March 1, 2020, and December 31, 2020, were included; pediatric and pregnant patients were excluded. Data collected included demographics, dates of hospital admission, ICU admission, hospital discharge, date of death, patients’ comorbidities, updated Charlson comorbidity index (CCI), presenting symptoms, vital signs upon admission, initial imaging, laboratory findings, need for intubation, and complications during the hospital stay. Data were collected using the electronic health information system at KAMC-Jeddah. Study subjects were dichotomized to poor and favorable outcomes. Poor COVID-19 outcome was defined as ICU admission, or death during hospital admission. This composite outcome was selected to include the hospital mortality events that occurred outside the ICU (emergency department and general wards) and to improve the statistical efficiency of the sample size.

The CCI is a validated score first introduced in 1984 and updated in 2011 (see Appendix). It assigns different weights to comorbid conditions and uses a cumulative score (0-24) to predict hospital mortality [14,15]. Inpatients with COVID-19 records were screened during their hospital stay for the development of the following complications: acute kidney injury, liver injury, acute cardiac injury, venous thromboembolism (VTE), and stroke (embolic, thrombotic, and venous sinus thrombosis). Acute kidney injury was diagnosed as an increase in serum creatinine by ≥26.5 µmol/L within 48 h or an increase in serum creatinine to ≥1.5 × baseline within a week [16]. The acute cardiac injury was defined as an elevated high-sensitivity troponin level above the 99th percentile of the upper reference range [17]. Liver injury was defined as alanine aminotransferase of at least three times the upper reference range or an increase in total bilirubin of at least twice the upper reference range [18]. Physician notes and medical imaging reports were used to document VTE and stroke.

The sample size was calculated using the formula N = 10 K/P, where K is the number of predictors needed in the model, and P is the smallest proportion of positive or negative cases. The formula yields a minimum of 10 events per predictive variable and accounts for the proportion of events within the study population. Accordingly, if approximately 20% of hospitalized patients with COVID-19 developed poor outcomes, a sample size of 400 is needed to predict the poor outcome using up to eight predictive variables [19].

Chi-square and Fisher’s exact tests were used to assess for an association between poor outcomes and the following variables: patients' characteristics (age, gender, obesity, updated CCI) and clinical features and complications of COVID-19. In addition, logistic regression analysis was performed to identify predictors of poor outcomes among patients with COVID-19.

## Results

Adult patients admitted in the general wards and ICUs with positive RT-PCR test results for COVID-19 between March 1, 2020, and December 31, 2021, at the KAMC-Jeddah comprised 527 patients. Forty-two patients (8%) (6-10, 95% confidence interval [CI]) died in total: 13 outside ICU and 29 in the ICUs. Of the 84 patients admitted to the ICU, 65 underwent invasive mechanical ventilation. Poor outcomes affected 97 patients (18%) (15-22, 95% CI). Mortality rates among patients admitted in the ICUs and general wards were 34% (29/84) and 3% (13/443), respectively. The median time to ICU admission was two days (interquartile range [IQR], 4), while the median time to death or hospital discharge was 20 days (IQR, 16) among the patients admitted to the ICU.

Regarding clinical features, shortness of breath and oxygen saturation <92% were associated with poor outcomes (P < 0.001), however; the total count of symptoms among patients with COVID-19 was not associated with poor outcomes (P = 0.9). Among patients with favorable and poor outcomes, the proportion of patients with abnormal initial chest x-ray was 207 out of 430 (49%), and 81 out of 97 (85%), respectively. A significant association was found between poor outcome and abnormal chest x-ray on admission (P < 0.001).  In addition, the total white blood cells count and c-reactive protein levels were significantly higher, while lymphocyte counts were significantly lower among patients with poor outcomes (P < 0.001).

The most common complications were acute cardiac injury (83 patients, 16%), acute kidney injury (78 patients, 15%), and liver injury (76 patients, 14%). On the other hand, VTE and cerebrovascular accidents affected 10 (2%) and five patients (1%), respectively (Table 1).

**Table 1:** Characteristics and complications of patients with COVID-19 ▫25th to 75th percentiles, *Mann-Whitney U test, •Charlson comorbidity index, ^^^Chi-square test, ^†^Fisher's exact test, ^‡^White blood cells and lymphocytes (×10^9^ g/L), ^∆^C-reactive protein (g/L).

Independent variables		n = 430 (%)	n = 97 (%)	P-value
		Favorable outcome	Poor outcome	
Age (years)	Q1-Q3▫	44-68	57-77	0.001*
Symptoms count	Q1-Q3▫	2-4	2-4	0.9*
Updated CCI• (0-24)	Q1-Q3▫	0-1	0-3	0.001*
Sex	Male	218 (80)	56 (20)	0.2^^^
	Female	212 (84)	41 (16)	
Obese (BMI ≥ 30)	No	206 (83)	43 (17)	0.6^^^
	Yes	224 (81)	53 (19)	
Shortness of breath	No	196 (90)	21 (10)	0.001^^^
	Yes	234 (75)	76 (25)	
Oxygen saturation < 92%	No	409 (84)	76 (16)	0.001^^^
	Yes	21 (50)	21 (50)	
Initial chest x-ray	Normal	216 (94)	14 (6)	0.001^^^
	Abnormal	207 (72)	81 (28)	
Venous thromboembolism	No	426 (83)	90 (17)	0.001^†^
	Yes	3 (30)	7 (70)	
Acute kidney injury	No	393 (88)	55 (12)	0.001^
	Yes	36 (46)	42 (54)	
Acute cardiac injury	No	392 (88)	52 (12)	0.001^†^
	Yes	38 (46)	45 (54)	
Liver injury	No	332 (83)	69 (17)	0.001^†^
	Yes	49 (64)	27 (36)	
Cerebrovascular accident	No	429 (82)	92 (18)	0.001^†^
	Yes	0 (0)	5 (100)	
Days to discharge or death	Q1-Q3▫	4-11	13-29	0.001*
	Median	7	20	
Total WBC^‡^	Q1-Q3▫	4-7	5-9	0.001*
Lymphocyte	Q1-Q3▫	1.0-1.8	0.7-1.4	0.001*
CRP^∆^	Q1-Q3▫	10-90	50-180	0.001*

Using multiple logistic regression, significant predictors for poor outcomes were the updated CCI (odds ratio [OR] 1.2 [95% CI 1.1-1.4]), liver injury (OR 2.6 [95% CI 1.3-4.9]), acute kidney injury (OR 4.3 [95% CI 2.3-7.8]), and acute cardiac injury (OR 5.1 [95% CI 2.8-9.4]) (Table 2). The only variable that had missing values >1% was liver injury (50/527) with 9% missing values. The effect of missing values on the results was checked using multiple imputations, and the results of the analysis of the original data and imputed data were similar. The results of the multiple imputation analysis were reported.

**Table 2:** Poor outcome predictors of patients with COVID-19 using multiple logistic regression OR, odds ratio; CI, confidence interval; BMI, body mass index; CCI, Charlson comorbidity index.

Predictors	Unadjusted			Adjusted		
	OR	P-value	95% CI	OR	P-value	95% CI
Female Sex	0.8	0.20	0.5-1.2	0.9	0.9	0.6-1.7
Age ≥65 years	2.6	0.001	1.7-4.0	1.2	0.6	0.7-2.1
Obese (BMI ≥ 30)	1.1	0.60	0.7-1.8	1.1	0.7	0.6-1.9
Updated CCI	1.4	0.001	1.2-1.5	1.2	0.003	1.1-1.4
Liver injury	2.2	0.004	1.3-3.8	2.6	0.004	1.3-4.9
Acute kidney injury	8.3	0.001	4.9-14	4.3	0.001	2.3-7.8
Acute cardiac injury	9.0	0.001	5.3-15	5.1	0.001	2.8-9.4

Data analysis was performed using the International Business Machine Statistical Package for the Social Sciences (IBM SPSS) Statistics version 28 (IBM, Armonk, New York, USA).

## Discussion

Poor outcome was observed in 18% (97/527) of patients, and the proportion of patients who died during admission was 8% (42/527). These findings are supported by previous national and international studies ranging from 6% to 33% for ICU admission and 6% to 14% for hospital mortality [11,12,20,21].

Several systematic reviews have shown that older age, obesity, and male sex increase the risk of poor outcomes among patients with COVID-19. The largest and most recent was a meta-analysis that included 42 studies with a total of 423,117 patients [20,22,23]. The model presented in the current study showed similar risk trends; however, the ORs for age, obesity, and sex were not significant. This is probably due to the smaller sample size of this cohort in comparison to the total sample size analyzed in the meta-analyses.

The CCI as a predictor of poor outcomes among patients with COVID-19 is supported by a meta-analysis of 20 studies and showed that higher CCI is associated with increased mortality among hospitalized patients with COVID-19 [24]. The updated CCI has a great advantage as it incorporates multiple comorbidities and age. The updated CCI OR was 1.2, meaning that among patients with similar COVID-19 complications, the odds of poor outcome increased on average by 20% for an updated CCI score of 1, and 44% (1.2^2^) for an updated CCI score of 2 [24,25].

A systematic review that included 49 studies identified injuries to the myocardium, kidneys, and liver as predictors of adverse outcomes with COVID-19 [26]. Several studies on COVID-19 patients yielded similar risk estimates for mortality in relation to acute cardiac injury (hazard ratio [HR] 1.6-7.2), acute kidney injury (OR 1.5-3.9), and liver injury (HR 1.2-2.3) [18,27,28].

Stroke and VTE were not included as predictors in the model owing to the low number of events. Five patients (1%) had a stroke, three ischemic, one hemorrhagic, and one cerebral sinus thrombosis. All patients with stroke were admitted to the ICU for mechanical ventilation, and two died. Two systematic reviews reported similar findings, with a stroke incidence of approximately 1% and an increased risk of poor outcomes [29,30]. 

Ten patients (2%) developed VTE, six of whom underwent mechanical ventilation, and two died. Patients included in this cohort were not screened routinely for VTE. Diagnostic imaging modalities were performed when VTEs were clinically suspected. In a systematic review that included 3,487 patients from 30 studies, the incidence of VTE varied from (0 to 60% among studies that used standard algorithms for clinically suspected VTE [31].

Lower lymphocyte count, higher neutrophils count, and higher c-reactive protein levels among patients with poor outcomes were documented in this study and reported by several other COVID-19 studies [20,32-35].

Poor outcomes were significantly associated with abnormal chest x-ray on admission. Similarly, previous studies showed that chest x-ray can help diagnose COVID pneumonia, predict poor outcomes, and monitor long-term complications of COVID-19 [34,36].

Finally, the results of this paper reflect the multisystemic nature of COVID-19 disease as the risk of poor outcomes is increased among patients with multiple comorbidities and/or multiple COVID-19 complications.

The strengths of this study are as follows. There were no eligibility restrictions regarding admitting patients with COVID-19; therefore, the cohort consisted of patients that represented most population sectors. An electronic health information system decreased the proportion of missing values among the model predictors to a minimum. This is the first study in Saudi Arabia to use the updated CCI to predict mortality in patients with COVID-19.

The limitations of this study included the following: the study was conducted in a single center, the data regarding D-dimer on admission was not collected, and subsequently it was not included in the analysis, and the chest computed tomography (CT) findings were not included as it was mainly done to patients who needed ICU. Therefore, using it to compare patients with poor and favorable outcomes was not feasible.

## Conclusions

COVID-19 disease is associated with significant morbidity and mortality. Predictors of poor outcomes among COVID-19 hospitalized patients were the updated CCI, liver injury, acute kidney, and acute cardiac injuries. Subsequently, the risk of poor COVID-19 outcomes is increased among patients with multiple comorbidities and/or multiple COVID-19 complications.

## Appendices

**Table 3:** Updated Charlson comorbidity index (CCI)* The following comorbid conditions were mutually exclusive: mild liver disease and moderate or severe liver disease; any malignancy and metastatic solid tumor. *Unofficial guide to orient study personnel about the updated CCI [15].

Comorbidities list	Updated weight
Myocardial infarction	0
Congestive heart failure	2
Peripheral vascular disease	0
Cerebrovascular disease	0
Dementia	2
Chronic pulmonary disease	1
Rheumatologic disease	1
Peptic ulcer disease	0
Diabetes without chronic complications	0
Diabetes with chronic complications	1
Hemiplegia or paraplegia	2
Renal disease	1
AIDS/HIV	4
Mild liver disease	2
Moderate or severe liver disease	4
Any malignancy, including leukemia and lymphoma	2
Metastatic solid tumor	6
Maximum comorbidity score	____ out of 24
